# When Too Much Fresh Air Is Bad for You: A Report of a Rare Case

**DOI:** 10.7759/cureus.79753

**Published:** 2025-02-27

**Authors:** João C Sousa, Maria I Risto, Catarina Trigo, Teresa Pinto

**Affiliations:** 1 Occupational Health, Hospital Santo António, Porto, PRT; 2 Internal Medicine, Hospital da Arrábida, Porto, PRT; 3 Internal Medicine, Centro Hospitalar Póvoa de Varzim/Vila do Conde, Vila do Conde, PRT; 4 Occupational Safety and Health, Serviço de Segurança e Saúde no Trabalho, ARS Norte IP, Porto, PRT

**Keywords:** farmer, occupational risk, pneumomediastinum, pneumorachis, spontaneous pneumomediastinum (spm)

## Abstract

Spontaneous pneumomediastinum (SPM) is a rare, non-traumatic accumulation of air in the mediastinum. Though typically benign, symptoms such as chest pain, dyspnea, and odynophagia require evaluation. Diagnosis involves imaging and laboratory findings to distinguish SPM from conditions requiring urgent intervention. Treatment is generally supportive, including oxygen therapy, analgesia, and observation, with invasive procedures considered only in cases of complications. This article presents the case of an 18-year-old male who developed chest pain and odynophagia following exertion. Imaging confirmed pneumomediastinum and pneumorachis. Managed conservatively, the patient fully recovered in eight days without recurrence. Recognizing occupational and lifestyle risks, particularly in strenuous activities, is essential for ensuring timely diagnosis and effective management.

## Introduction

Spontaneous pneumomediastinum (SPM) is a clinical condition characterized by the presence of air or gas within the mediastinum in the absence of detectable traumatic injury or underlying lung disease [1]. While a rare condition with an estimated occurrence of about 1 to 3 cases per 100,000 individuals annually, identification and familiarity with SPM are important because it has a unique clinical presentation, poses difficulties in diagnosis, and has special management implications [2]. Demographically, SPM primarily affects young, otherwise healthy individuals, with a higher incidence in males, most prominently among those aged 20 to 30 years [3].

SPM is most frequently caused by strenuous physical exertion, asthma exacerbations, severe coughing, vomiting, the Valsalva maneuver, and aspiration [2]. The presentation typically includes chest pain, dyspnea, odynophagia, and subcutaneous emphysema [3]. A prompt diagnosis should be established because SPM shares clinical features with other potentially life-threatening conditions, such as pneumothorax and esophageal rupture, which should be ruled out immediately to avoid misdiagnosis and improper management [4].

Diagnostic evaluation typically involves radiographic imaging, and the imaging modality of choice is chest X-ray and computed tomography (CT). Chest X-ray is typically the first imaging modality due to its availability and efficacy in detecting pneumomediastinum. However, CT is the gold standard for confirming the diagnosis, as it provides a clearer and more accurate picture of the extent of air in the mediastinum and can also identify associated complications such as pneumothorax or esophageal rupture [1,3]. Laboratory findings, such as mild leukocytosis and neutrophilia, may provide corroborative evidence but are not diagnostic on their own [5,6].

Management of SPM is usually conservative, with admission recommended for monitoring, oxygen therapy, and symptomatic management. As most cases are self-limiting, the primary goal is to monitor for complications and keep the patient comfortable. Oxygen therapy, which increases the resorption of free air in the mediastinum, can be used to accelerate recovery and reduce symptoms [2]. Symptomatic management includes pain relief and monitoring for the appearance of complications, such as hypertensive pneumomediastinum or mediastinitis, which require immediate intervention [4,7].

## Case presentation

An 18-year-old Caucasian male farmer, with a BMI of 24, presented with no significant personal or family medical history, no known allergies, and no regular medication use. His national vaccination schedule was up to date. He arrived at the emergency department complaining of odynophagia and thoracic pain, which had persisted for approximately 24 hours. The day before, the patient had engaged in moderate-intensity physical activity on his farm, transferring straw bales weighing up to 20 kg. No trauma was reported.

**Figure 1:**
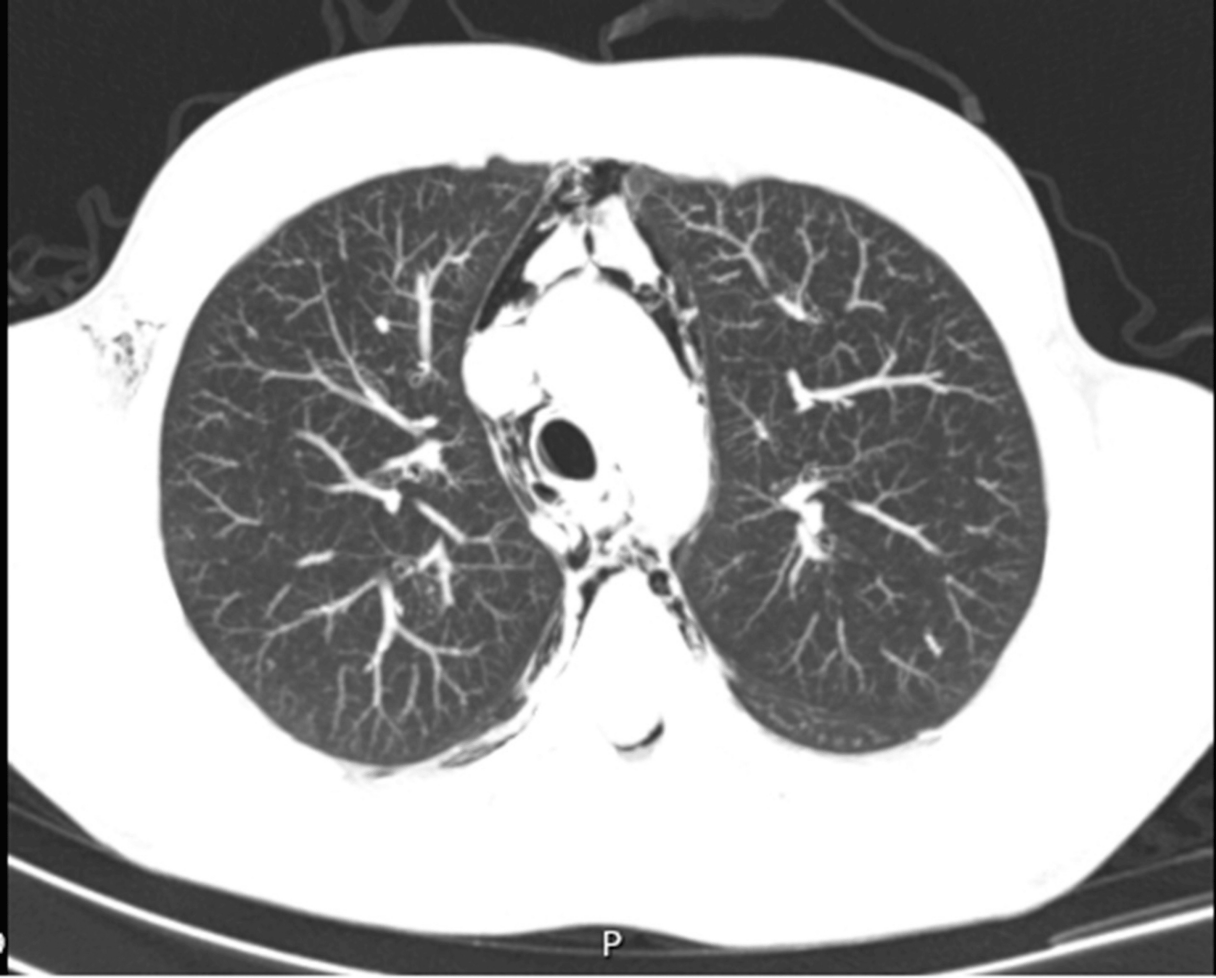
CT scan revealing pneumomediastinum.

On admission, the patient was afebrile, hemodynamically stable, and showed no signs of respiratory distress. Physical examination revealed a general state of good health. Initial laboratory tests (Table 1), arterial blood gases (Table 2), and electrocardiogram results were all within normal limits. Chest radiography revealed signs of mediastinal enlargement, prompting a chest CT scan. The CT scan confirmed pneumomediastinum, with air extending into the neck and vertebral canal, affecting the spinal cord and posterior intervertebral foramina (Figure 1). No pneumothorax or parenchymal abnormalities were noted. The patient was started on oxygen therapy and admitted to an intermediate care unit for monitoring.

**Table 1:** Laboratory results.

Parameter	Result	Reference Values
Hemoglobin	15.1 g/dL	13.2-17.2 g/dL
Leukocytes	7.72 x 10^9^/L	4 x 10^9^/L to 11 x 10^9^/L
Platelets	226 x 10^9^/L	150 x 10^9^/L to 400 x 10^9^/L
C-reactive protein	2.7mg/L	<3.0 mg/L
AST (aspartate aminotransferase)	21 U/L	10-32 U/L
ALT (alanine aminotransferase)	25 U/L	10-33 U/L
LDH (lactate dehydrogenase)	165 U/L	135-225 U/L
Gama GT (gamma-glutamyltransferase)	34 U/L	6-42 U/L
AP (alkaline phosphatase)	58 U/L	35-105 U/L
Creatinine	0.89 mg/dL	0.51-0.95 mg/dL
Urea	29 mg/dL	10-50 mg/dL
INR (International Normalized Ratio)	0.9	0.8-1.2
PT (prothrombin time)	12.1 s	11.5-14.5 s

**Table 2:** Arterial blood gases upon admission to the emergency department.

Parameter	Result	Reference Values
pH	7.40	7.35-7.45
pO2	98 mmHg	60-100 mmHg
pCO2	37 mmHg	35-45 mmHg
Lactates	0.6 mmol/L	<2.5 mmol/L

The patient was treated with high-flow oxygen and analgesics. On the eighth day of hospitalization, a follow-up chest CT revealed complete resolution of pneumomediastinum and pneumorachis. He was followed up in the Internal Medicine outpatient department and remained asymptomatic. Three months post-discharge, he returned to work with restrictions, not lifting heavy loads. Six months later, he was on all duties without any restrictions. One year after discharge from the clinic, a follow-up CT scan revealed normal results, signifying full recovery.

## Discussion

SPM is usually self-limiting and benign but requires careful evaluation to rule out severe differential diagnoses, such as pneumothorax or esophageal rupture [2,4]. It typically results from alveolar rupture due to increased intrathoracic pressure, with consequent air dissection along the bronchovascular sheath into the mediastinum [1].

Imaging is the cornerstone of diagnosis, with chest X-ray as the first-line modality and CT as the more sophisticated assessment [3]. The "ring-around-the-artery" sign and air tracking along the bronchovascular bundle are the hallmarks [4,6]. Laboratory investigations, although non-specific, can show mild leukocytosis and neutrophilia [5].

Treatment is still fundamentally conservative, consisting of oxygen therapy, analgesia, and close observation [1,2]. Oxygen therapy plays an important role in the rapid reabsorption of free air from the mediastinum, as a higher concentration of oxygen accelerates the absorption of oxygen and nitrogen from interstitial fluid. It not only helps resolve pneumomediastinum but also alleviates pain and dyspnea. Prophylactic antibiotics remain highly controversial, and most cases resolve spontaneously within one week [3].

Hospitalization is usually recommended for patients with moderate to severe symptoms, such as significant chest pain, dyspnea, or subcutaneous emphysema. For severe dyspnea, hemodynamic instability, or suspected complications like pneumothorax or esophageal rupture, hospitalization becomes necessary for close observation and immediate intervention when needed. Observational management in an outpatient setting may be acceptable for patients presenting with mild symptoms and no comorbid risk factors [4]. Invasive procedures, such as chest tube insertion, are reserved for complications like tension pneumomediastinum [4].

Occupational hazards, particularly those associated with physically demanding jobs, warrant special attention [5]. Manual labor involving heavy lifting may predispose individuals to SPM due to increased intrathoracic pressure [2,8]. Preventive measures, such as proper lifting techniques and awareness of breathing, can minimize the risk [3,6].

## Conclusions

Despite the complete recovery of the patient, it is important to bear in mind that SPM recurrence can be high in certain high-risk populations. Those with physically demanding jobs, such as laborers who carry heavy loads, may be at increased risk due to greater intrathoracic pressure. Also, patients with pre-existing respiratory conditions, such as asthma or chronic obstructive pulmonary disease (COPD), may be at greater risk of recurrence. In such patients, long-term plans of care should include preventive measures such as proper lifting techniques, respiratory conditioning, and close follow-up for early recurrence detection. The absence of recurrence does not guarantee the elimination of future episodes, particularly when risk factors exist.
